# Lhermitte-Duclos Disease in an Eight-Year-Old Boy: A Case Report

**DOI:** 10.7759/cureus.62076

**Published:** 2024-06-10

**Authors:** Omar M Alawaji, Ghassan M Aweja

**Affiliations:** 1 Neurological Surgery, King Fahad Hospital, Medina, SAU; 2 Neurosurgery, Aseer Central Hospital, Abha, SAU

**Keywords:** lhermitte-duclos disease, cowden syndrome, pediatric neurosurgery, hamartomatous malformation, lhermitte-duclos

## Abstract

Lhermitte-Duclos disease (LDD), also known as dysplastic cerebellar gangliocytoma, is a rare, slow-growing, benign lesion that occurs in the cerebellum and is very uncommon in the pediatric population. There is a lack of literature and evidence about LDD management, and only one systematic review is available. Thus, more case reports and studies are warranted. This study reports a pediatric case diagnosed with LDD and describes the patient’s clinical presentation, radiological findings, and histopathological criteria. In addition, important aspects of the disease are discussed to help reach the best management options. The main management option is surgical resection, though a "wait and see" approach is also an alternative, especially for asymptomatic patients. More studies are still needed to determine the best management options.

## Introduction

Lhermitte-Duclos disease (LDD) is a benign hamartomatous malformation that is rare in young adults and very rare in children, with only a few case reports having been published. It is also known as cerebellar gangliocytoma and was first introduced by Lhermitte and Duclos in 1920 [1]. It is characterized by slow abnormal growth in the cerebellum and affects the granular cells. According to the World Health Organization (WHO), it is categorized as a grade-1 tumor [2].

The worldwide incidence of LDD is unknown, but over 300 cases have been reported in the literature since its first description. The most common age group is 20-40 years, and there is no gender predominance [3]. The most common location is the cerebellum, but Bevan et al. and Azzarelli et al. reported rare and unique occurrences in the hypothalamus and the spinal cord. However, only one case reported has described bilateral occurrence in the cerebellar hemispheres in a patient with Cowden syndrome [4].

The diagnosis of LDD is done using radiological imaging and histopathology results, including MRI. The lesion is hypointense in T1-weighted imaging and shows a “tiger-stripe” pattern of alternating high and low intensity levels in T2-weighted imaging [5]. Pathologically, it is characterized by thickening of the molecular and granular cell layers of the cerebellar cortex [6].

The management of LDD has not been well studied. It has been established that a "wait and see" approach is acceptable with serial images and observation until the condition becomes symptomatic. The relevant symptoms include high intracranial pressure, any brain-stem compression, or fourth-ventricle compression causing obstructive hydrocephalus. Upon the appearance of such symptoms, surgery and resection are performed. Some surgeons offer surgery even for asymptomatic patients for the purpose of both treating and diagnosing the lesion. There are very few case reports on pediatric LDD. Thus, we report a case in a young and otherwise healthy boy to help improve the understanding of this rare disease in this rarer age group.

## Case presentation

An eight-year-old boy came to our emergency department for a life-saving referral from a peripheral facility. He had a three-day history of headache and repeated vomiting, as well as one episode of generalized tonic colonic convulsion, which lasted for 10 minutes, stopped spontaneously, and was followed by postictal confusion and headache. A neurological examination showed only an unsteady gait but was unremarkable otherwise. Another systemic review was performed to look specifically for skin manifestation and criteria for Cowden syndrome, but the result was completely unremarkable. The patient was otherwise healthy.

**Figure 1:**
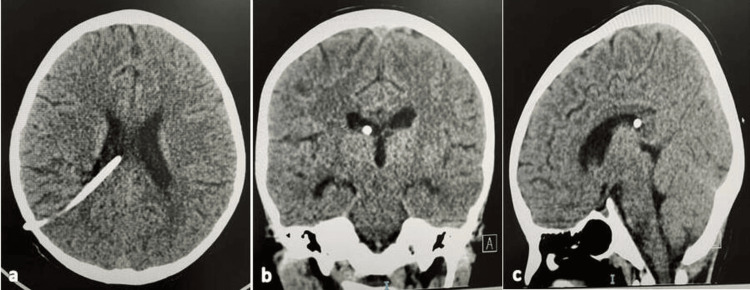
Brain CT images of an eight-year-old boy with Lhermitte-Duclos disease (LDD) after right-side ventriculoperitoneal shunt insertion (a) Axial view; (b) Coronal view; (c) Sagittal view

An urgent CT scan was performed and showed supra-tentoria hydrocephalus with non-specific poorly differentiated anatomy of the left cerebellum. An urgent overnight ventriculoperitoneal shunt with a medium pressure valve was inserted in the right parietal lobe, and the operation was smooth with no complications during or after the operation (see Figure 1). On the second day, the patient was doing fine, fully conscious, and communicating, with only a mild headache.

**Figure 2:**
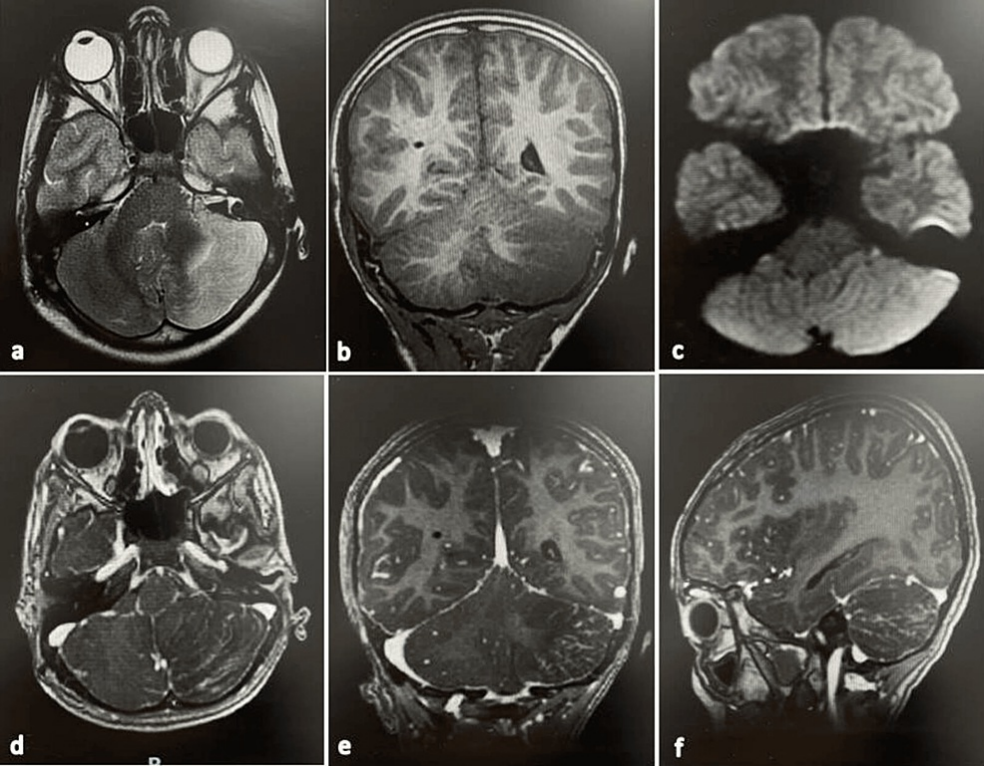
Pre-operative brain MRI of an eight-year-old boy with Lhermitte-Duclos disease (LDD) (a) Axial T2-weighted image showing patchy high-signal lesion in the left cerebellum with mass effect and compression of the fourth ventricle. The classic tiger-stripe appearance is considered typical for LDD; (b) Coronal T1-weighted image showing low-signal lesion in the left cerebellum (typical finding); (c) Axial diffusion weighted imaging (DWI) showing high signal (restricted diffusion); (d–f) Axial, coronal, and sagittal contrasted T1-weighted images.

Contrast-enhanced cranial MRI was performed done and showed an ill-defined left-cerebellar lesion with patchy high signals both in T2 and flair imaging and low signals in T1 imaging with restriction in diffusion weighted imaging (DWI). No definite mass noted with contrast enhancement following sulci distribution (see Figure 2). Furthermore, the DWI sequence did not show any diffusion restriction. The potential differential diagnoses were LDD, viral infection, acute ischemic insult, and leukemia. In the next four days, the patient did fine with no complaints and was prepared for partial resection operation.

At seven days post admission, the patient was transferred to the operation room, and a partial resection was performed using a left sub-occipital approach. Almost 30% of the lesion was removed, and there were no complications during or after the operation. After opening the dura, an almost normal cerebellar parenchyma was observed with somewhat stiff and very well-vascularized tissue, but there was no difficulty during resection. The reason for performing the partial resection was to confirm the diagnosis and to avoid sacrificing the whole left cerebellar hemisphere for such a benign lesion. After three days, the patient was discharged home. He still had unsteady gait as he did preoperatively, which showed only mild improvement.

**Figure 3:**
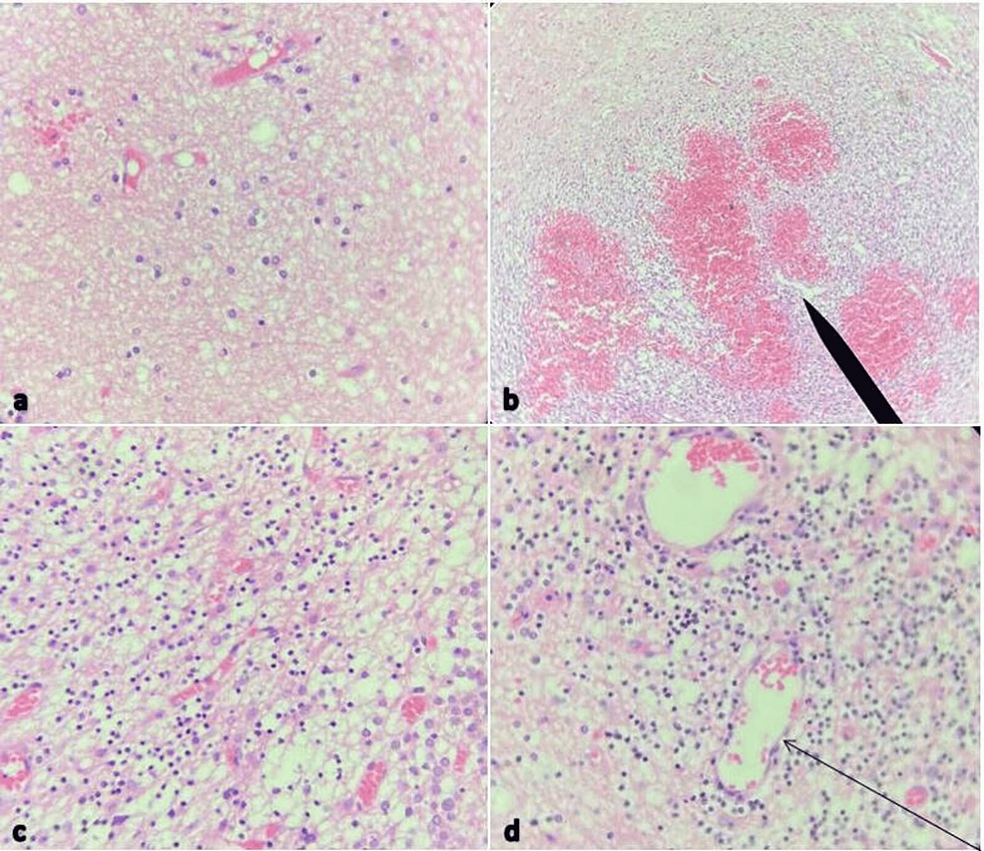
Photomicrographs of a pathological slide of an 8-year-old boy with Lhermitte-Duclos disease (LDD) (a) Specimen showing variable mixture of dysplastic ganglion cells with different sizes; (b) Specimen showing prominent capillary network (arrow); (c) Specimen showing ganglion-like cells exhibiting binucleation, cytomegaly with ballooning cytoplasm, and lack of organized cytoarchitecture; (d) Specimen showing perivascular lymphoid

The histopathology results (see Figure 3) showed that the lesion had a variable mixture of dysplastic ganglion cells with different sizes (Figure 3a). The glial cells lacked atypical neoplastic characteristics (hypercellularity, hyperchromatic nuclei, necrosis, etc.). A prominent capillary network was observed (Figure 3b), and the ganglion-like cells exhibited binucleation, cytomegaly with ballooning cytoplasm, and lack of organized cytoarchitecture (Figure 3c). Perivascular lymphoid infiltrates were present (Figure 3d), and there was no mitotic activity. During the follow-up period, the wound healed with no scar, the patient’s unsteady gait improved dramatically, and he had no other complaints. The results of examinations were completely normal. We planned to perform MRI follow-up at 6, 12, 18, and 48 months since there was no literature to support any other plan.

## Discussion

LDD is a hamartomatous malformation in the cerebellum, and case reports of this condition are rare. It usually occurs in the second to fourth decades of life, but our patient was an eight-year-old male. Patients are usually asymptomatic and follow-up with serial images is preferred once the diagnosis is confirmed.

Once the patient becomes symptomatic with high intracranial pressure or vital structure compression, debulking is advisable. Headache and ataxia are the most common presentations [7]. Hydrocephalus (as in our case) is a rare presentation that has been reported in less than 16% of published cases [7]. Somagawa et al. reported a case with repeated vomiting without high intracranial pressure signs or symptoms, which was explained by a mass effect in the area postrema in the medulla oblongata [8].

LDD is commonly associated with some syndromes, and the most common one is Cowden syndrome. This condition is characterized by criteria that were described by the international Cowden Syndrome Consortium in 2015, which include multiple skin hamartomas and different neoplasms [9]. However, none of these criteria were found in the present case.

The radiology criteria of the lesion include non-specificity in CT scans and sometimes calcification. In MRI, hypointensity occurs in T1-weighted sequences with no contrast enhancement, while characteristic hyperintense striated appearance occurs in the T2 weighted sequence [10]. This classic tiger-stripe appearance has been described in more than of 58% of cases, but it does not always occur [7]. For example, Ezgu et al. reported a case with no obvious striated pattern [11].

The main management options are surgical resection (all types), gross-total resection (GTR), subtotal resection (STR), and partial resection (as in our case). Very good results have been reported in more than 60% of the reported cases [7]. Recurrence after resection is common in patients who have other conditions such as Cowden syndrome. Other options such as chemotherapy and radiotherapy need to be studied more to determine their relevance in LDD. Zak et al. reported a patient treated with rapamycin therapy, which showed good outcomes [12]. Conservative management using a "wait and see" approach is also an acceptable option as the disease is slow growing with no invasion characteristics.

The differential diagnosis is very important in these cases as LDD can mimic some high-grade lesions, such as medulloblastoma, which has the same characteristic appearance in MRI (as in our case with restricted diffusion) [13-15]. Another differential diagnosis is pseudotumoral hemicerebellitis, which has been described in three pediatric cases reported by Bosemani et al. [16]. Thus, it was necessary to confirm our diagnosis using histopathology results.

## Conclusions

LDD is a rare disease and is even rarer in pediatric populations. Presentations differ, although headache and ataxia are the most common symptoms. Knowing the criteria for Cowden syndrome is important for the workup, follow-up and prognosis. Radiology criteria are important but do not rule out other important differential diagnoses. Histological analysis is mandatory for all cases before a management decision is established. The main management is surgical resection, although a "wait and see" approach is an acceptable alternative, especially for asymptomatic patients.

More studies and systemic reviews must be done to address important questions related to LDD. For example, it is not clear whether one can rely on radiology to diagnose LDD and whether there is a risk for high-grade transformation. Furthermore, the best management options must be determined, and non-surgical options should be explored. Questions also remain in regard to the risk of recurrence, the growth rate of lesions, specific criteria for applying a "wait and see" approach, and the best steps for follow-up plans.
